# Kynurenic Acid and Its Analog SZR104 Exhibit Strong Antiinflammatory Effects and Alter the Intracellular Distribution and Methylation Patterns of H3 Histones in Immunochallenged Microglia-Enriched Cultures of Newborn Rat Brains

**DOI:** 10.3390/ijms23031079

**Published:** 2022-01-19

**Authors:** Melinda Szabo, Noémi Lajkó, Karolina Dulka, István Szatmári, Ferenc Fülöp, András Mihály, László Vécsei, Karoly Gulya

**Affiliations:** 1 Department of Cell Biology and Molecular Medicine, University of Szeged, 6720 Szeged, Hungary; szabo.melinda.1@med.u-szeged.hu (M.S.); lajko.noemi@med.u-szeged.hu (N.L.); dulka.karolina@med.u-szeged.hu (K.D.); 2 ELKH–SZTE Stereochemistry Research Group, Institute of Pharmaceutical Chemistry, University of Szeged, 6720 Szeged, Hungary; szatmari.istvan@szte.hu (I.S.); fulop.ferenc@szte.hu (F.F.); 3 Interdisciplinary Excellence Center, Institute of Pharmaceutical Chemistry, University of Szeged, 6720 Szeged, Hungary; 4 Department of Anatomy, University of Szeged, 6720 Szeged, Hungary; mihaly.andras@med.u-szeged.hu; 5 ELKH–SZTE Neuroscience Research Group, Department of Neurology, Interdisciplinary Excellence Center, University of Szeged, 6720 Szeged, Hungary; vecsei.laszlo@med.u-szeged.hu

**Keywords:** antiinflammation, CCR1, CXCL10, cytoplasmic histone, H3K9me3, H3K36me2, kynurenic acid, SZR104

## Abstract

Kynurenic acid (KYNA) is implicated in antiinflammatory processes in the brain through several cellular and molecular targets, among which microglia-related mechanisms are of paramount importance. In this study, we describe the effects of KYNA and one of its analogs, the brain-penetrable SZR104 (N-(2-(dimethylamino)ethyl)-3-(morpholinomethyl)-4-hydroxyquinoline-2-carboxamide), on the intracellular distribution and methylation patterns of histone H3 in immunochallenged microglia cultures. Microglia-enriched secondary cultures made from newborn rat forebrains were immunochallenged with lipopolysaccharide (LPS). The protein levels of selected inflammatory markers C–X–C motif chemokine ligand 10 (CXCL10) and C–C motif chemokine receptor 1 (CCR1), histone H3, and posttranslational modifications of histone H3 lys methylation sites (H3K9me3 and H3K36me2, marks typically associated with opposite effects on gene expression) were analyzed using quantitative fluorescent immunocytochemistry and western blots in control or LPS-treated cultures with or without KYNA or SZR104. KYNA and SZR104 reduced levels of the inflammatory marker proteins CXCL10 and CCR1 after LPS-treatment. Moreover, KYNA and SZR104 favorably affected histone methylation patterns as H3K9me3 and H3K36me2 immunoreactivities, and histone H3 protein levels returned toward control values after LPS treatment. The cytoplasmic translocation of H3K9me3 from the nucleus indicated inflammatory distress, a process that could be inhibited by KYNA and SZR104. Thus, KYNA signaling and metabolism, and especially brain-penetrable KYNA analogs such as SZR104, could be key targets in the pathway that connects chromatin structure and epigenetic mechanisms with functional consequences that affect neuroinflammation and perhaps neurodegeneration.

## 1. Introduction

Several recent experimental and clinical studies have found that endogenous tryptophan metabolites, including kynurenic acid (KYNA), are involved in several neurophysiological and neuropathophysiological mechanisms [1,2,3]. The biological significance of the kynurenine pathway; KYNA synthesis, degradation, and excretion; and the kynurenine pathway’s immunomodulatory properties in vertebrates are all well established [2,3,4,5]. In fact, KYNA exerts modulatory effects on the immune system through the regulation of T cells, natural killer cells [4], and microglial cells [5]. The neuroprotective role of KYNA in different inflammatory/neurodegenerative central nervous system disorders is of particular interest. However, since KYNA does not pass the blood–brain barrier, researchers are attempting to synthesize KYNA analogs that can penetrate this barrier [6] and hence to provide possible treatments for neurodegenerative or neuroinflammatory disorders [7,8]. One such analog, N-(2-(dimethylamino)ethyl)-3-(morpholinomethyl)-4-hydroxyquinoline-2-carboxamide (SZR104; Table S1), was recently synthesized [9,10] and applied successfully against pentylenetetrazole-induced seizures, and in doing so significantly it decreased the seizure-evoked field potentials [11]. Moreover, KYNA and SZR104 exhibit antiinflammatory properties both in vitro and in vivo; they markedly inhibit the lipopolysaccharide (LPS)-stimulated phagocytotic activity of cultured microglial cells and thus display potent immunosuppressive capabilities in an animal model of epilepsy [5].

Microglial cells are the intrinsic immune cells of the central nervous system, and they possess complex cellular and molecular mechanisms that detect deviations from homeostasis in nervous tissue. Under physiological conditions, microglia are mostly ramified and survey the parenchymal integrity of the central nervous system [12]. At the site of damage or inflammation, activated microglia change their morphology, express increased levels of major histocompatibility antigens, and become phagocytic [13]. They also release inflammatory cytokines and other potentially cytotoxic substances that can amplify inflammatory responses by activating and recruiting other cells to a lesion or infection [14,15]. For example, inflammation can be exacerbated by the secretion of the C–X–C motif chemokine ligand 10 (CXCL10) from microglia [16,17] or other cell types [18,19,20]. CXCL10 induces chemotaxis, apoptosis, the inhibition of cell growth, and angiostasis [21]. Both CXCL10 and its receptor (the C–X–C motif chemokine receptor 3) are crucial for leukocyte trafficking and homing to inflamed tissues, as well as for the perpetuation of inflammation that leads to tissue damage [22]. Previous studies have reported that CXCL10 is involved in the pathophysiology of multiple sclerosis [1,23]. Similarly, the C–C motif chemokine receptor 1 (CCR1) and its ligands may play a role in the pathogenesis of multiple sclerosis [24].

DNA in the nucleus is wrapped around proteins known as histones, which form the chromatin structure. The capability of eukaryotic cells to maintain their diverse phenotypes is ensured by the chemical modifications of the DNA molecule, the activities of chromatin-associated proteins, and numerous posttranslational modifications of the histone proteins [25,26,27]. Although histones are typically located inside the nucleus, where they regulate transcription, they are known to have a wide range of functions in various cellular and extracellular locations as well [25,26,27]. When they are in the extracellular milieu, they become damage-associated molecular patterns that promote inflammation, cytotoxicity, coagulation, and apoptosis [28,29,30,31]. Cytoplasmic functions of histone proteins include participation in cell signaling pathways related to the mediation of immunological functions such as innate immunity [28]. The cytoplasmic accumulation of histones and nucleosomes precedes the externalization of phagocytosis signals on the outer membrane surface of apoptotically dying lymphoblasts [32,33,34]. For instance, the translocation of a specific histone H1 subtype from the nucleus into the cytoplasm triggers the release of cytochrome C from the mitochondria and thus leads to apoptosis [35].

Histone modifications (acetylations, methylations, phosphorylations, ubiquitinations, etc.) are posttranslational modifications made in the nucleus by the appropriate enzymes [25,32,36]. As a consequence, transcription often becomes altered because modified histones and the DNA will interact differently. The cytoplasmic accumulation of such modified histones might indicate that they were released/transported from the nucleus, perhaps as a consequence of distress [29,30,31]. Histone modifications are key epigenetic regulatory features that govern many cellular functions. Specific histone posttranslational modifications can direct site-specific activation or the silencing of transcription [36]; hence, they are the principal players that regulate gene expression. Histone methylations at lysine (lys (K)) and arginine (arg (R)) residues are relatively stable and considered potential marks for carrying the epigenetic information present in specific regions of the genome. For example, several monomethylations (i.e., H3K9me1, H3K27me1, and H3K79me1) and some dimethylations of histone H3 proteins (H3K36me2) are linked to active transcription, while other dimethylations (H3K9me2) and most trimethylations of this core histone (i.e., H3K9me3, H3K27me3, and H3K79me3) are linked to gene repression [37,38].

In this study, we investigated the relationships among (a) KYNA and its analog SZR104, (b) the inflammatory mechanisms that gives rise to epigenetic changes via histone methylations, and (c) the intracellular localizations of unmethylated and methylated histones in microglial cells. Besides the inflammatory markers CXCL10 and CCR1, we quantitatively analyzed the levels of unmodified core histone H3 and histone H3 lys methylations at the H3K9me3 and H3K36me2 sites (Table S2), marks that are considered contrary in regulating gene expression [37,38] and also involved in immunomodulation [39,40,41,42], using western blots and multicolor light microscopic immunofluorescence. As far as we know, our approach for studying KYNA and its brain-penetrable analog SZR104, with regard to epigenetics and neuroinflammation, is unique in the literature. Our results shed light on the indicator roles that these histones, translocated to the cytoplasm, might play in neuroinflammation; furthermore, our findings highlight the beneficial role that the endogenous kynurenine system could play in antiinflammatory mechanisms.

## 2. Results

### 2.1. KYNA and SZR104 Downregulate LPS-Induced CXCL10 Levels While Differentially Altering CCR1 Levels

**Figure 1:**
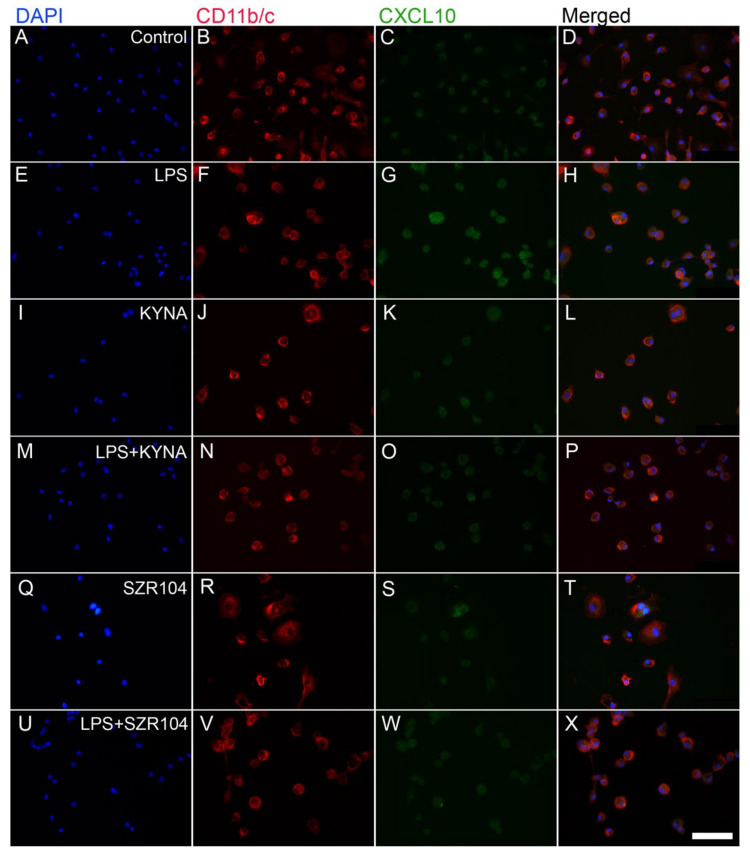
Localization of CXCL10 immunoreactivity in CD11b/c-labeled microglial cells in unchallenged and treated microglia-enriched cultures. The distribution of CD11b/c (red) and CXCL10 (green) immunoreactivities and their colocalizations is shown. The anti-CD11b/c antibody was used to highlight microglial cells. Note the very high purity of the microglial cultures (DAPI vs. CD11b/c labels). The following cultures (subDIV7) were used: (**A**–**D**) unstimulated (control), (**E**–**H**) LPS-challenged, (**I**–**L**) KYNA-treated, (**M**–**P**) LPS + KYNA-treated, (**Q**–**T**) SZR104-treated, and (**U**–**X**) LPS + SZR104-treated cultures. Cell nuclei are labeled with DAPI (blue). CXCL10 immunoreactivity was more intensive after LPS treatment in microglia; KYNA and SZR104 decreased the amount of CXCL10 in these cells. No visible cell loss was observed after the treatments were applied. This is in agreement with the findings of Steiner et al. [43], who found there was no effect on cell viability when microglial cells were treated with KYNA. LPS: 20 ng/mL; KYNA: 1 µM; and SZR104: 1 µM. Scale bar: 75 µm.

In CD11b/c-labeled microglial cells taken from unchallenged (control) and treated microglia-enriched secondary cell cultures (subDIV7), there was a robust increase in the amount of immunoreactivity of the inflammation marker CXCL10 in LPS-treated microglia (Figure 1). Following an LPS immunochallenge, typical ameboid morphology was observed (Figure 1F) with CXCL10 immunoreactivity localized in the cytoplasm (Figure 1G).

**Figure 2:**
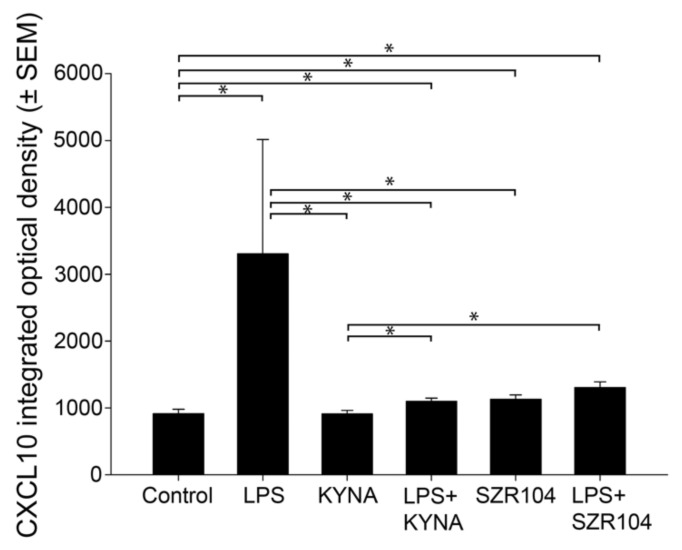
Quantitative light microscopic microdensitometric analysis of CXCL10 protein expression in unchallenged and treated microglia-enriched cultures. The LPS challenge significantly elevated cytoplasmic CXCL10 immunoreactivity (approximately fourfold) in microglial cells, whereas KYNA alone, SZR104 alone, or the combined treatments significantly weakened the CXCL10 immunoreactive signal to levels observed in unchallenged (control) cells. LPS: 20 ng/mL; KYNA: 1 µM; and SZR104: 1 µM. Data (presented as means ± SEMs) were analyzed using Kruskal–Wallis one-way ANOVA on ranks: * *p* < 0.05.

**Figure 3:**
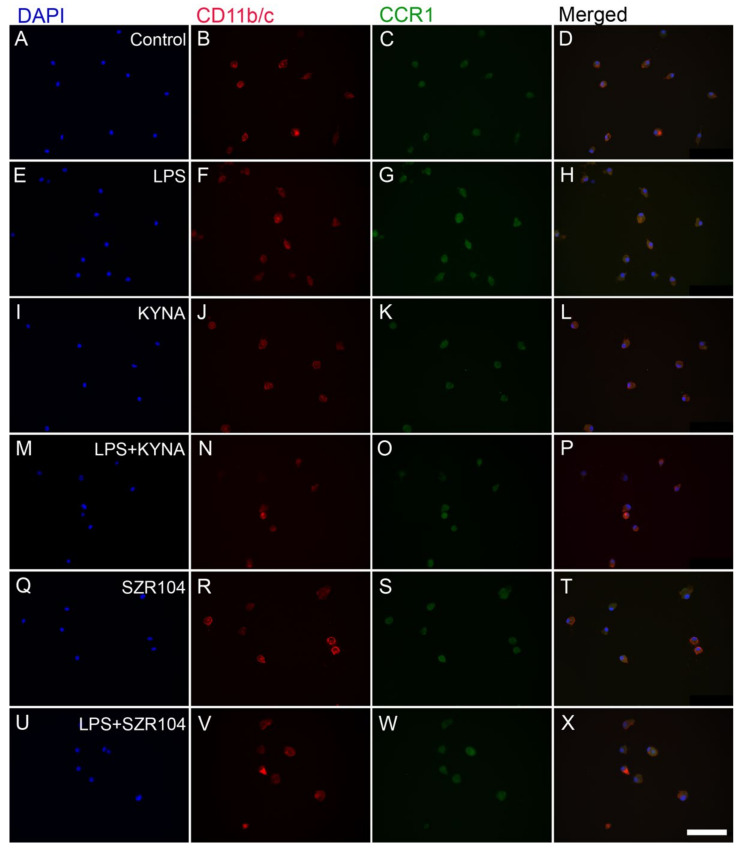
Localization of CCR1 immunoreactivity in CD11b/c-labeled microglial cells in unchallenged and treated microglia-enriched cultures. The distribution of CD11b/c (red) and CCR1 (green) immunoreactivities, as well as their colocalizations, is shown. The anti-CD11b/c antibody was used to highlight microglial cells. Note the very high purity of the microglial cultures (DAPI vs. CD11b/c labels). The following cultures (subDIV7) were used: (**A**–**D**) unstimulated (control), (**E**–**H**) LPS-challenged, (**I**–**L**) KYNA-treated, (**M**–**P**) LPS + KYNA-treated, (**Q**–**T**) SZR104-treated, and (**U**–**X**) LPS + SZR104-treated cultures. Representative immunocytochemical images confirm that the LPS challenge (**G**) slightly increased CCR1 immunoreactivity in microglial cells compared with that in unchallenged (control) cells (**C**), but the level of the immunoreactive signal returned to control levels with KYNA (**K**), SZR104 (**S**), or combined treatments (**O**,**W**). LPS: 20 ng/mL; KYNA: 1 µM; and SZR104: 1 µM. Scale bar: 75 µm.

**Figure 4:**
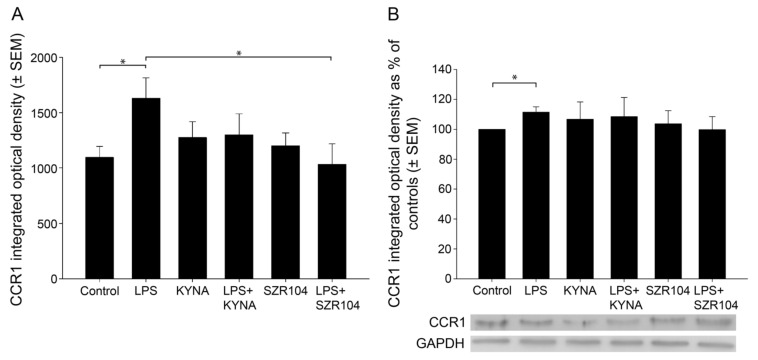
Quantitative analysis of CCR1 protein expression in unchallenged and treated microglia-enriched cultures. (**A**) A quantitative light microscopic microdensitometric analysis of CCR1 protein expression. The LPS challenge significantly elevated CCR1 immunoreactivity to approximately 148% of the control value in microglial cells, whereas KYNA or SZR104 alone, or the combined treatment of LPS + KYNA, did not significantly alter the amount of CCR1 immunoreactive signal compared to controls. However, LPS + SZR104-treated cultures displayed significantly lowered CCR1 levels compared to LPS-treated cultures, and they returned to levels seen in unchallenged (control) cells. Data (presented as means ± SEMs) were analyzed with the Mann–Whitney rank sum test: * *p* < 0.05. (**B**) A quantitative western blot analysis of cytoplasmic CCR1 immunoreactivity. Representative images of western blots are shown below the graph, together with the GAPDH immunoreactive bands that served as protein load control. Protein samples were collected from at least five separate cultures, electrophoresed, and then quantitatively analyzed, as described in the Materials and Methods section. CCR1 immunoreactivity significantly increased after the LPS treatment. It did not change when the cultures were treated with LPS + KYNA or LPS + SZR104. Error bars indicate integrated optical density values with the data values for each group expressed as a percentage of the control values. LPS: 20 ng/mL; KYNA: 1 µM; and SZR104: 1 µM. Integrated optical density data (presented as means ± SEMs) were analyzed with the Mann–Whitney rank sum test: * *p* < 0.02.

Treatments with KYNA or SZR104 (a brain-penetrable KYNA analog), either alone or in combination with LPS, reduced CXCL10 immunoreactivity to unchallenged levels (Figure 1K,O,S,W). Moreover, quantitative light microscopic microdensitometric analysis of CXCL10 protein expression revealed that LPS challenge significantly elevated CXCL10 immunoreactivity (approximately fourfold) in microglial cells, whereas KYNA alone, SZR104 alone, or either combined with LPS significantly depleted the CXCL10 immunoreactive signal to control (unchallenged) levels (Figure 2). A similar but weaker response was recorded when the immunoreactivity of CCR1, another inflammation marker, was tested in control and treated cells. A localization analysis in CD11b/c-labeled microglial cells revealed slightly increased CCR1 immunoreactivity in LPS-challenged cultures (Figure 3G) relative to that observed in the control (Figure 3C). A quantitative light microscopic microdensitometric analysis of the cellular CCR1 levels of the cultures showed that an LPS challenge significantly elevated CCR1 immunoreactivity by 48%, whereas KYNA or SZR104 alone, or the combined treatment with LPS + KYNA, proved ineffective (Figure 4A). Interestingly, the combined treatment of LPS + SZR104 significantly lowered CCR1 immunoreactivity as compared to LPS-treated cultures, and it reverted to a level observed in unchallenged (control) cultures. A quantitative western blot analysis revealed that cytoplasmic CCR1 immunoreactivity was significantly increased after LPS treatment (Figure 4B). KYNA or SZR104, either alone or in combination with LPS, did not noticeably affect CCR1 levels.

### 2.2. KYNA and SZR104 Alter the Intracellular Histone H3 Distribution and H3 lys Methylation Patterns

**Figure 5:**
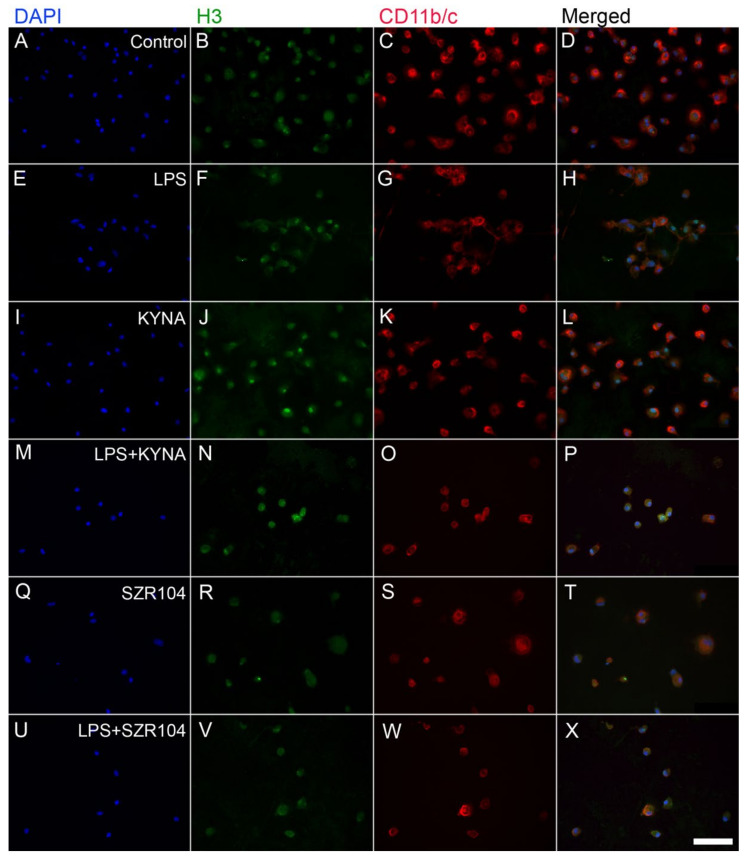
Localization of histone H3 protein immunoreactivity in unchallenged and treated microglia-enriched cultures. Representative immunocytochemical images demonstrate the intracellular distribution of histone H3 protein immunopositivity (green) in unstimulated (control) (**A**–**D**), LPS-challenged (**E**–**H**), KYNA-treated (**I**–**L**), LPS + KYNA-treated (**M**–**P**), SZR104-treated (**Q**–**T**), and LPS + SZR104-treated (**U**–**X**) CD11b/c-immunopositive microglial cells (red). The anti-CD11b/c antibody was used to highlight microglial cells. Note the very high purity of the microglial cultures (DAPI vs. CD11b/c labels). Histone H3 was detected in both the nucleus and cytoplasm of microglia. Cell nuclei are labeled with DAPI (blue). LPS: 20 ng/mL; KYNA: 1 µM; and SZR104: 1 µM. Scale bar: 75 µm.

**Figure 6:**
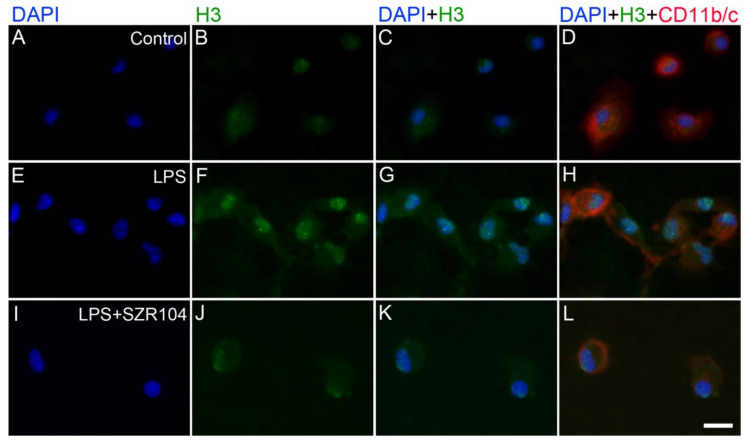
Intracellular localization of histone H3 protein immunoreactivity in unchallenged and treated microglia-enriched cultures. Representative enlarged immunocytochemical images showing a subset of microgllial cells from Figure 5 demonstrate the intracellular distribution of histone H3 immunopositivity (green) in unstimulated (control) (**A**–**D**), LPS-challenged (**E**–**H**), and LPS + SZR104-treated (**I**–**L**) CD11b/c-immunopositive microglial cells (red). The anti-CD11b/c antibody was used to highlight microglial cells. After LPS treatment (**F**), unmodified histone H3 was detected in both the nucleus and cytoplasm of microglia. LPS + SZR104 treatments lowered both nuclear and cytoplasmic H3 immunosignal. Cell nuclei are labeled with DAPI (blue). Scale bar: 15 µm.

**Figure 7:**
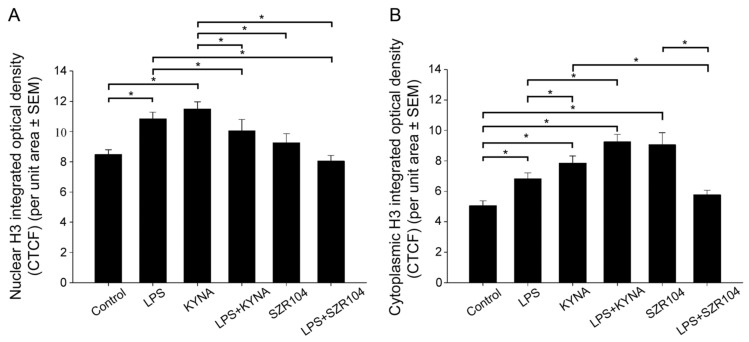
Intracellular distribution of unmodified histone H3 protein immunoreactivity in the nucleus and cytoplasm of microglia in unchallenged and treated microglia-enriched cultures. Corrected total cell fluorescence (CTCF) values for the whole cell, nucleus, and cytoplasm were calculated as described in the Materials and Methods section. (**A**) The amount of H3 immunoreactivity rose significantly in the nucleus of LPS- and KYNA-treated microglia. SZR104 effectively decreased the amount of histone H3 after LPS treatment. (**B**) Except for the LPS + SZR104 treatment, all the treatments increased the amount of unmodified cytoplasmic histone H3. LPS: 20 ng/mL; KYNA: 1 µM; and SZR104: 1 µM. Data (presented as means ± SEMs) were analyzed with Kruskal–Wallis one-way ANOVA on ranks: * *p* < 0.05.

Unmodified histone H3 levels were monitored because they form a pool for further posttranslational modifications. H3 immunoreactivity was detected in both the cytoplasm and nucleus of unchallenged microglia; that is, after nuclear import, histone H3 mostly accumulated in the nucleus (Figure 5A–D). We also did not detect any extracellular histone signal in these studies. Interestingly, every experimental manipulation of the cells, except for the LPS + SZR104 treatments, resulted in the increased accumulation of histone H3 in both the nuclear and the cytoplasmic compartments, indicating that both elevated de novo synthesis and increased nuclear import occurred as outcome of the treatments (Figure 5E–X and Figure 6F). Of these treatments, KYNA produced the strongest nuclear accumulation of unmodified H3 immunoreactivity (Figure 5J). When LPS and SZR104 treatments were combined, however, neither the nuclear nor the cytoplasmic H3 immunoreactivities were different from the controls (Figure 6). Quantitative microdensitometry of H3 immunosignals on cultured microglia corroborated these findings (Figure 7A). The CTCF values for nuclear localization increased significantly only after LPS or KYNA treatments, compared to controls, but decreased significantly after the combined treatments of LPS + KYNA and LPS + SZR104, as compared to LPS-challenged cultures (Figure 7A). In contrast, CTCF values for cytoplasmic localization were higher in all experimental groups except for the LPS + SZR104 treatment case (Figure 7B). Moreover, cytoplasmic H3 levels were affected differently by the combined treatments, i.e., LPS + KYNA increased, while LPS + SZR104 returned cytoplasmic H3 levels to controls (Figure 6J and Figure 7B). The nuclear and cytoplasmic histone H3 distributions elicited by the LPS + SZR104 treatment were rather like those of unchallenged microglia.

**Figure 8:**
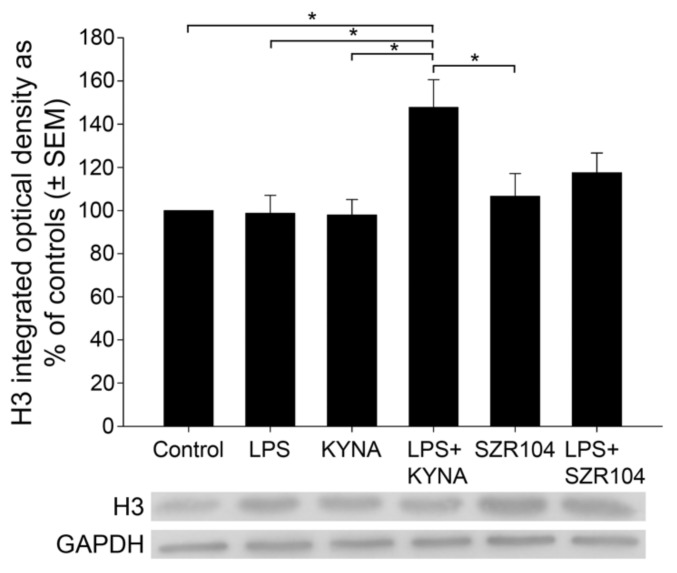
Quantitative western blot analysis of the cytoplasmic histone H3 protein level in microglia-enriched cultures. Representative images of western blots are shown below the graph, together with the GAPDH immunoreactive bands that served as inner standards. Protein samples were collected from at least five separate cultures (subDIV7), electrophoresed, and quantitatively analyzed as described in the Materials and Methods section. The combined LPS + KYNA treatment induced a significant increase in histone H3 immunoreactivity when compared with that in control (unchallenged) and other treated cultures. The error bars indicate integrated optical density values with data expressed as a percentage of the control values. LPS: 20 ng/mL; KYNA: 1 µM; and SZR104: 1 µM. Data values (presented as means ± SEMs) were analyzed using ANOVA followed by pairwise multiple comparisons (Holm–Sidak method): * *p* < 0.05.

However, when the amount of unmodified cytoplasmic histone H3 was quantitatively analyzed via western blots, only the LPS + KYNA treatment showed a significant increase (Figure 8). This might be due to denaturing conditions in detecting histone H3 proteins in westerns that concealed the changes observed in multicolor immunocytochemistry when individually identified microglia were analyzed.

**Figure 9:**
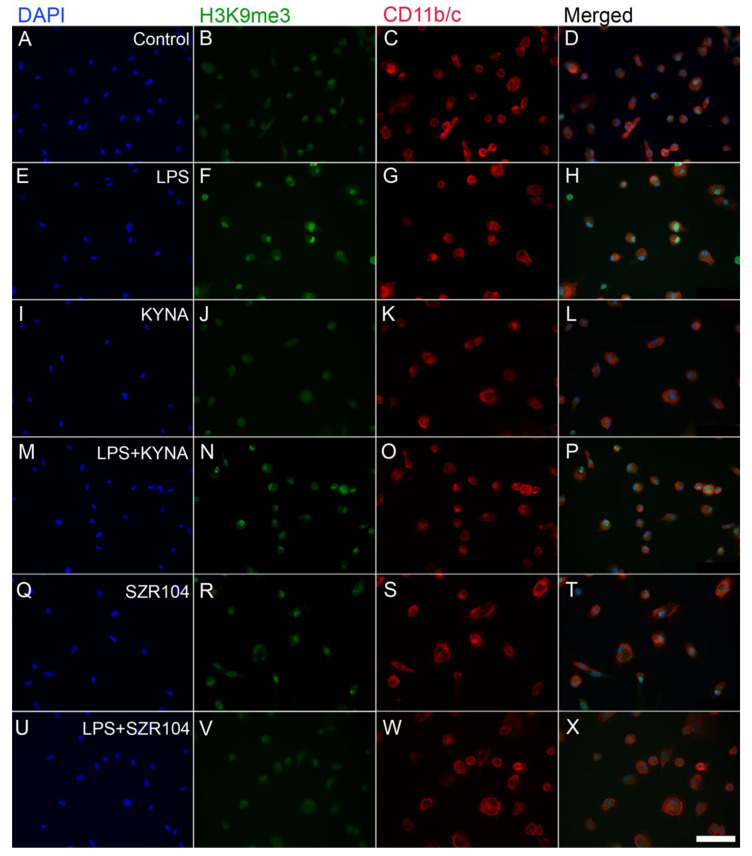
Localization of H3K9me3 immunoreactivity in CD11b/c-labeled microglia in unchallenged and treated microglia-enriched cultures. Representative immunocytochemical images showing the intracellular distribution of histone H3K9me3 protein immunopositivity (green) in unstimulated (control) (**A**–**D**), LPS-challenged (**E**–**H**), KYNA-treated (**I**–**L**), LPS + KYNA-treated (**M**–**P**), SZR104-treated (**Q**–**T**), and LPS + SZR104-treated (**U**–**X**) CD11b/c-immunopositive microglial cells (red). The anti-CD11b/c antibody was used to highlight microglial cells. The very high purity of the microglial cultures is evident (DAPI vs. CD11b/c labels). Note that LPS challenge (**F**) increased the H3K9me3 immunopositivity relative to that in unchallenged controls (**B**) or other treatments. Histone H3K9me3 was detected in both the nucleus and cytoplasm. LPS: 20 ng/mL; KYNA: 1 µM; and SZR104: 1 µM. Scale bar: 75 µm.

**Figure 10:**
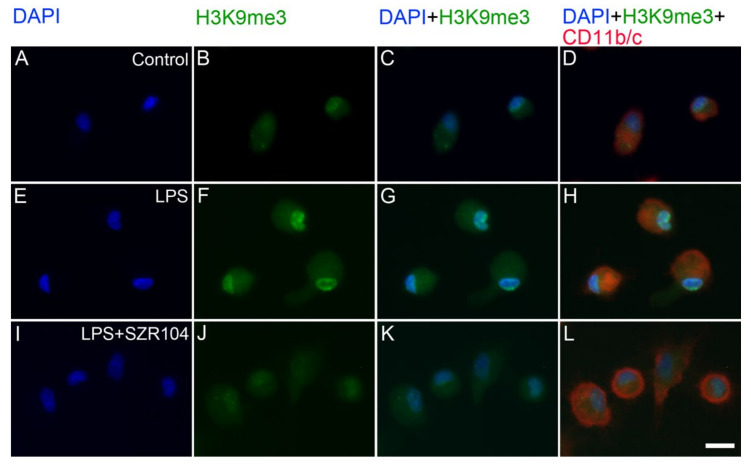
Intracellular localization of H3K9me3 immunoreactivity in CD11b/c-labeled microglia in unchallenged and treated microglia-enriched cultures. Representative enlarged immunocytochemical images showing a subset of microgllial cells from Figure 9 demonstrate the intracellular distribution of histone H3K9me3 immunopositivity (green) in unstimulated (control) (**A**–**D**), LPS-challenged (**E**–**H**), and LPS + SZR104-treated (**I**–**L**) CD11b/c-immunopositive microglial cells (red). The anti-CD11b/c antibody was used to highlight microglial cells. After LPS treatment (**F**), increased amounts of H3K9me3 immunolabel were detected in both the nucleus and cytoplasm of microglia, although the nuclear component was more pronounced. LPS + SZR104 treatments lowered the amounts of both nuclear and cytoplasmic H3K9me3 immunosignal. Cell nuclei are labeled with DAPI (blue). Scale bar: 15 µm.

**Figure 11:**
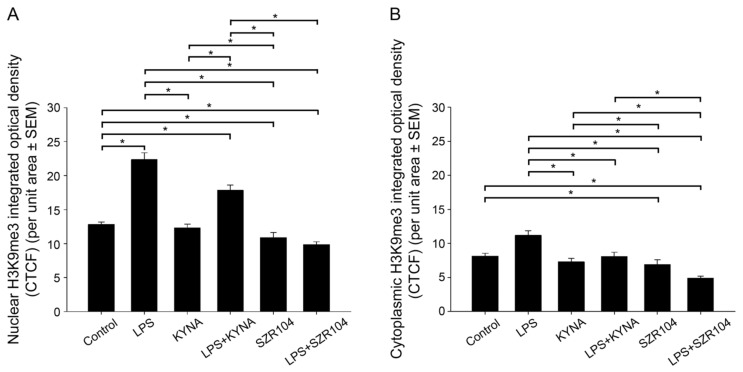
Intracellular distribution of histone H3K9me3 protein immunoreactivity in the nucleus and cytoplasm of microglia in unchallenged and treated microglia-enriched cultures. A quantitative microdensitometric analysis of H3K9me3-immunopositive signals in the nucleus (**A**) and cytoplasm (**B**) was performed, as described in the Materials and Methods section. (**A**) LPS treatment increased the amount of H3K9me3 signal in the nucleus, whereas KYNA and SZR104 treatment did not alter the signal compared to the control level. (**B**) Cytoplasmic H3K9me3 was reduced uniformly when cells were treated with KYNA, SZR104, or with a combination of treatments. LPS: 20 ng/mL; KYNA: 1 µM; and SZR104: 1 µM. Integrated density data values (presented as means ± SEMs) were analyzed with Kruskal–Wallis one-way ANOVA: * *p* < 0.05.

Our analysis of the intracellular localization of H3K9me3 immunoreactivity in CD11b/c-labeled microglia in unchallenged (control) and variably treated microglia-enriched secondary cell cultures (subDIV7) demonstrated that LPS challenge (Figure 9E–H, Figure 10F) increased H3K9me3 immunpositivity relative to that observed in the unchallenged control (Figure 9A–D and Figure 10B) or other treatments (Figure 9I–X). Strong histone H3K9me3 immunoreactivity was detected in both the nucleus and cytoplasm after the LPS challenge (Figure 9F and Figure 10F), but decreased when LPS treatment was combined with KYNA or SZR104 (Figure 10J). Quantitative fluorescent microdensitometry revealed that the nuclear accumulation of H3K9me3 protein increased significantly after LPS treatment but that the LPS + KYNA or LPS + SZR104 treatments reduced this accumulation (Figure 10J and Figure 11A). However, the effect of KYNA alone did not differ significantly from that of the control. By contrast, SZR104 had a greater inhibiting effect on the extranuclear translocation of H3K9me3. Similar values and tendencies were noted when cytoplasmic H3K9me3 was analyzed (Figure 11B): KYNA or SZR104, either alone or in combination with LPS, was able to recover LPS-induced cytoplasmic accumulation of H3K9me3 protein. However, SZR104 was found to be more potent than KYNA in inhibiting H3K9me3 translocation to the cytoplasm (Figure 11B).

**Figure 12:**
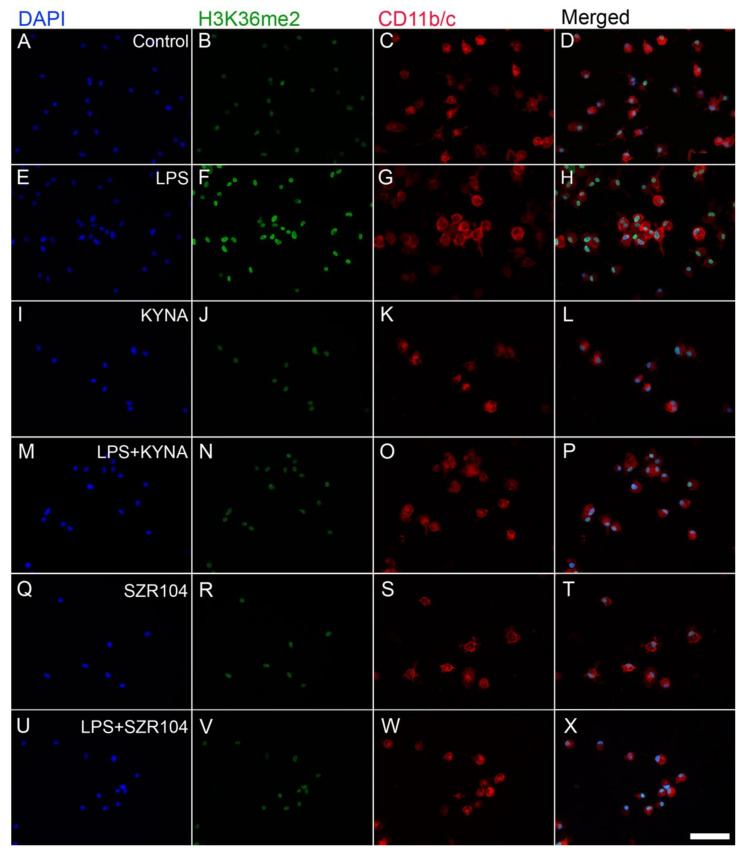
Localization of H3K36me2 immunoreactivity in CD11b/c-labeled microglia in unchallenged and treated microglia-enriched cultures. Representative immunocytochemical images showing the intracellular distribution of histone H3K36me2 protein immunopositivity (green) in unstimulated (control) (**A**–**D**), LPS-challenged (**E**–**H**), KYNA-treated (**I**–**L**), LPS + KYNA-treated (**M**–**P**), SZR104-treated (**Q**–**T**), and LPS + SZR104-treated (**U**–**X**) CD11b/c-immunopositive microglial cells (red). The anti-CD11b/c antibody was used to highlight microglial cells. The very high purity of the microglial cultures is evident (DAPI vs. CD11b/c labels). Note that the LPS challenge (**E**–**H**) markedly increased H3K36me2 immunopositivity relative to that of the unchallenged control (**A**–**D**) or any other treatment (**I**–**X**). Histone H3K36me2 was detected in the nucleus. LPS: 20 ng/mL; KYNA: 1 µM; and SZR104: 1 µM. Scale bar: 75 µm.

**Figure 13:**
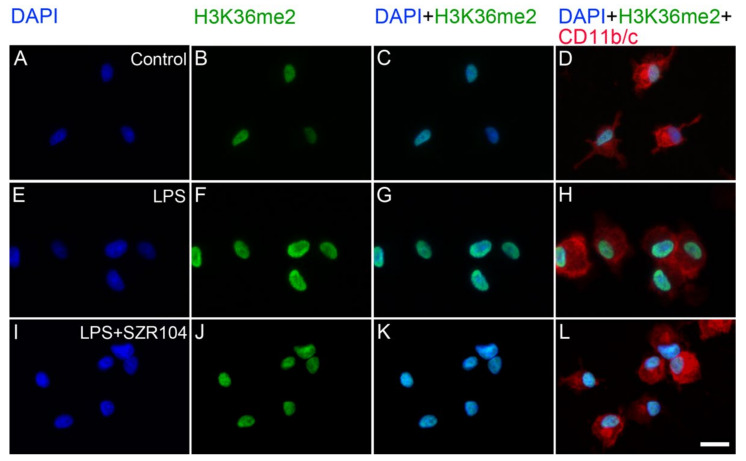
Intracellular localization of H3K36me2 immunoreactivity in CD11b/c-labeled microglia in unchallenged and treated microglia-enriched cultures. Representative enlarged immunocytochemical images showing a subset of microgllial cells from Figure 12 demonstrate the intracellular distribution of histone H3K36me2 immunopositivity (green) in unstimulated (control) (**A**–**D**), LPS-challenged (**E**–**H**), and LPS + SZR104-treated (**I**–**L**) CD11b/c-immunopositive microglial cells (red). The anti-CD11b/c antibody was used to highlight microglial cells. After LPS treatment (**F**), increased amounts of H3K36me2 immunolabel were detected in the nucleus of microglia. There was no signal in the cytoplasm. LPS + SZR104 treatments lowered the amounts of nuclear H3K36me2 immunosignal (**J**). Cell nuclei are labeled with DAPI (blue). Scale bar: 15 µm.

**Figure 14:**
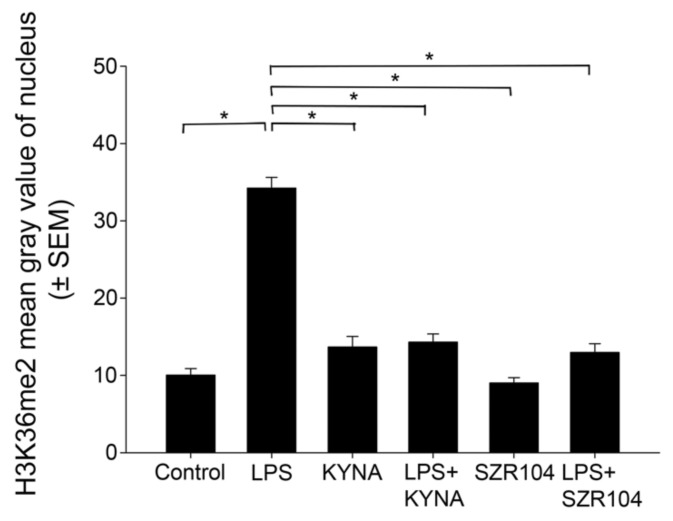
Intracellular distribution of histone H3K36me2 protein immunoreactivity in the nucleus of microglia in unchallenged and treated microglia-enriched cultures. A quantitative microdensitometric analysis of H3K36me2-immunopositive signals in the nucleus was performed as described in the Materials and Methods section. The LPS treatment dramatically increased the amount of the H3K36me2 signal in the nucleus, whereas KYNA, SZR104, or a combination of treatments decreased it toward the unchallenged control levels. LPS: 20 ng/mL; KYNA: 1 µM; and SZR104: 1 µM. The integrated density data values (presented as means ± SEMs) were analyzed with Kruskal–Wallis one-way ANOVA on ranks: * *p* < 0.05.

When the intracellular distribution of H3K36me2 immunoreactivity in CD11b/c-labeled microglia was measured, a marked increase, relative to unchallenged control levels (Figure 12A–D), was seen exclusively within the nuclei of the microglia after LPS challenge (Figure 12E–H and Figure 13F). By contrast, the other treatments did not affect H3K36me2 immunoreactivity appreciably (Figure 12I–X). For example, LPS + SZR104 treatments returned the nuclear levels of H3K36me2 to the control levels (Figure 13J). A quantitative microdensitometric analysis of H3K36me2-immunopositive signals revealed that LPS treatment dramatically increased the amount of H3K36me2 signal in the nuclei of CD11b/c-labeled microglial cells, whereas the other treatments had only a minor effect. Moreover, KYNA or SZR104 alone, or the combination treatments, had significantly lower H3K36me2 signals compared to LPS-challenged levels (Figure 14).

## 3. Discussion

In the functioning of the immune system, endogenous kynurenine metabolism is implicated. Recent studies showed that the amounts of several inflammation-related marker proteins decreased after treatments with KYNA or its analogs. For example, it was demonstrated by Mándi et al. [8] and Lajkó et al. [5] that KYNA and several KYNA analogs, including SZR104, interfered with immune functions in vivo and in vitro. KYNA and SZR104 not only attenuated tumor necrosis factor-α (TNF-α) production and increased tumor necrosis factor-stimulated gene-6 mRNA expression in U-937 cells stimulated with heat-inactivated *Staphylococcus aureus* [8] but they also inhibited the LPS-stimulated phagocytotic activity of microglial cells in vitro while suppressing microglial activity in an in vivo model of epilepsy [5]. Another potent proinflammatory citokine, interleukin-1β, was elevated in sepsis but was ameliorated by KYNA and its synthetic analogues SZR72 and SZR104 [44]. Furthermore, SZR72 inhibited the production of the inflammatory mediators TNF-α, calprotectin, S100A12, and HNP1-3 in blood cultures of rheumatoid arthritis patients [45]. Interestingly, a possible role for indoleamine 2,3-dioxygenase, a key kynurenine pathway enzyme, in immunity has recently emerged [46], further emphasizing the crucial role this system plays in immunomodulatory functions. As KYNA is a metabolite of the endogenous kynurenine system, with proven antiinflammatory properties, we wanted to know whether its effect went beyond conventional targets in the intermediary metabolic or intracellular signaling pathways and perhaps had effects on phenomena such as epigenetics elicited through histone metabolism/transport. In particular, we sought to investigate (a) how SZR104, a brain-penetrable analog of KYNA, behaves in our systems; (b) how inflammatory signals affect histone methylations and, consequently, epigenetic changes; and (c) how the intracellular localization of unmodified and methylated histones change during inflammation or amelioration of inflammation.

In the present study, KYNA and SZR104 exhibited strong antiinflammatory properties, as demonstrated by their effective amelioration of LPS-challenged CXCL10 and CCR1 production in microglial cultures. This in vitro functional study shows for the first time that KYNA and SZR104 effectively ameliorate the production of two proinflammatory signal molecules, CXCL10 and CCR1, in LPS-challenged microglia. Our results are in agreement with those of other studies that found similar CXCL10 reduction in activated microglia after treatment with antiinflammatory drugs [47,48]. Earlier, we previously reported a similar downregulation of CCR1 after aspirin treatment in LPS-challenged microglial-enriched cultures [49]. Structural differences between KYNA and SZR104 might be the reason these compounds produced somewhat different responses in immunocytochemical and western blot analyses. While KYNA and SZR104 were both effective in ameliorating the LPS-induced elevation of CXCL10 immunoreactivity, only the combined treatment with LPS + SZR104 was effective in significantly inhibiting CCR1 immunoreactivity in these tests. As we used high-purity microglia, this was also the first time that we could identify microglia-specific immune responses to these compounds.

The strong antiinflammatory action of KYNA and SZR104 on inflammatory marker proteins was accompanied by a similarly favorable regulation of histone methylation marks in microglia-enriched cultures. We demonstrated that KYNA and SZR104 reverted the H3K9me3 and H3K36me2 immunoreactivities toward levels observed in the control, i.e., unchallenged values, after immunochallenge by LPS treatment. Our findings also indicated that the cytoplasmic translocation of methylated H3K9 proteins from the nucleus after the LPS challenge was a cellular response to immunological distress. Cytoplasmic translocation from the nucleus of these methylated histones could be ameliorated or inhibited by KYNA and SZR104, which confirms the antiinflammatory nature of these drugs in the present experimental setup. We also observed differential effects of KYNA and SZR104 on the cytoplasmic H3 localization; that is, LPS + KYNA increased, while LPS + SZR104 returned cytoplasmic H3 levels to control values. The detailed mechanisms behind these differential effects of KYNA and SZR104 are not yet understood.

Histones are essential structural and functional components of chromatin. Histone proteins are typically located in the nucleus, but they have functions at extranuclear or even extracellular sites. For instance, extracellular histones released in response to a bacterial challenge contribute to endothelial dysfunction, renal failure and death during sepsis [50]. Patients with sepsis have increased levels of extracellular histones that are correlated with a poor prognosis and the development of sepsis-related consequences such as end-organ damage. These histones originate in megakaryocytes that contain cytoplasmic histones and transfer the proteins to their platelet progeny [31]. Observations on cytoplasmic accumulation of histones have been made in certain pathologic states. For example, altered and differential intracellular histone distribution were detected by Wu et al. [51]. They showed that not all cell lines released histones from nucleosomes during DNA fragmentation and apoptosis, while Gabler et al. [32] demonstrated that the cytoplasmic accumulation of histones and nucleosomes in physiological cells was a precursor to apoptosis, occurring in parallel with the initial phagocytosis signals. During microglia activation by LPS, for instance, DNA damage and genome instability were observed [52,53]. Unmodified H2B in the cytoplasm acts as a sensor that detects double-stranded DNA fragments derived from infectious agents or damaged cells and as a consequence activates innate and acquired immune responses in various cell types [54,55]. Therefore, the cytoplasmic localization of histones is also of pathophysiological importance. For example, the translocation of nuclear histone H1 to the cytoplasm in cultured pulmonary arterial smooth muscle cells is associated with pathologic states such as idiopathic pulmonary hypertension [56]. The cytoplasmic accumulation of the unmodified nucleosomal histones H1, H2A, H2B, H3, and H4 in cell lysates was observed very early on in the process of apoptosis [32,34]. When the effects of doxorubicin, an anthracycline widely used in anticancer therapy, were tested on the aggregation and intracellular distribution of both partners of the H2A-H2B dimer, marked differences between the two histones were found [57]. For H2A, aggregation retention was observed; for H2B, a massive accumulation in the cytoplasm of Jurkat leukemia cells was observed concomitant with its disappearance from the nuclei.

Alterations in histone posttranslational modifications are viewed as an important process by which various cellular functions, including transcription [25,58], gene silencing [59], and immunity [60], are regulated. For example, methylation sites can influence the binding of epigenetic factors to histone tails, which alters the extent to which DNA is wrapped around histone proteins and the availability of genes in the DNA to be activated [38,61,62]. In neuronal cultures, Hayakawa et al. [63] found three metabolites (kynurenine, 3-OH-kynurenine, and anthranilate) from the tryptophan pathways that increase H3K4 trimethylation, resulting in upregulated gene expression at hippocampal-linked loci (except those encoding pan-neural markers). Dimethylated and trimethylated H3K9 sites, i.e., transcriptionally repressive marks, are both found more often at silenced genes [37] and are typical of heterochromatic regions [64]. For instance, H3K9me2 is important in the regulation of inflammatory responses because it suppresses interferon and interferon-inducible antiviral gene expression [65] and epigenetically attenuates target gene-induction by inflammatory signaling in vascular smooth muscle cells [66]. H3K9me3 has been implicated in the opening of chromatin on inflammatory gene promoters, and it is seen at significantly increased levels in treatment-resistant tumors [67]. Additionally, macrophages cultured in high-glucose conditions display increased expressions of cytokine genes and decreased H3K9me3 levels when compared with cells incubated in a normal glucose culture [68]. Methylation of H3K36 has also been found to be related to inflammatory functions and transcription of proinflammatory genes [39,40,41]. Our data suggest that while the expressions of unmodified histone H3 proteins and inflammatory marker proteins such as CXCL10 and CCR1 are probably regulated independently from each other by pro- and antiinflammatory agents, the subcellular localization of this protein and its methylated forms could be affected by both pro- and antiinflammatory agents through yet unidentified mechanisms.

In summary, methylations of the histone H3 lys sites seem to be essential epigenetic marks for inflammation. Interestingly, KYNA and its analog SZR104 might act on KYNA signaling pathways that potentially ameliorate neuroinflammation through the elicitation of antiinflammatory actions. Our findings corroborate previous studies on the antiinflammatory properties of endogenous KYNA and raise the possibility that some of the newly designed KYNA analogs that can penetrate the blood–brain barrier may alter gene expression epigenetically to activate antiinflammatory mechanisms. Hence, our findings may lead to the development of antiinflammatory medications targeting the central nervous system.

## 4. Materials and Methods

### 4.1. Animals

All our animal experiments were conducted in strict compliance with the European Council Directive (86/609/EEC) and EC regulations (O.J. of EC No. L 358/1, 18/12/1986), related to the care and use of laboratory animals for experimental procedures, and the relevant Hungarian and local legislation requirements were followed. Experimental protocols were approved by the Institutional Animal Welfare Committee of the University of Szeged (II./1131/2018; date of approval: 30 May 2018). The pregnant Sprague–Dawley rats (190–210 g) used in this study were maintained under standard housing conditions and fed ad libitum. A total of five breeding runs (with 5–7 pregnant rats each) provided the litters (6–12 pups from each mother), from which independent culturing experiments were performed.

### 4.2. Reagents and Antibodies

KYNA (mol. weight: 189.17 g) was purchased from Sigma-Aldrich (Budapest, Hungary), and SZR104 (mol. weight: 358.43 g; Table S1) was synthesized in-house as described in previous studies by our laboratories [9,10,69]. KYNA and SZR104 were dissolved in Dulbecco’s Modified Eagle’s Medium (DMEM; Invitrogen, Carlsbad, CA, USA) and added at the appropriate concentration to the cultures. Bacterial lipopolysaccharide (LPS; Sigma-Aldrich) was used to elicit immunochallenge. The primary and secondary antibodies used in our study are listed in Table S2. For the characterization of microglial cells, we used an antibody against the CD11b/c, clone OX-42 [70]. We also used antibodies against the secreted ligand CXCL10 and the receptor CCR1 as inflammation markers [21,71]. In addition, we used antibodies against the unmodified core histone H3 protein and its posttranslational modifications at lys sites, H3K9me3, and H3K36me2, to detect the cytoplasmic and nuclear localizations of these proteins. An anti-glyceraldehyde 3-phosphate dehydrogenase (GAPDH) antibody was used as an internal control in western blot experiments [72].

### 4.3. Cell Culture

Forebrain tissue samples taken from newborn Sprague–Dawley rats of both sexes were removed; cleared from the meninges; minced with scissors; and homogenized for 10 min at 37 °C in DMEM containing 1 g/L D-glucose, 110 mg/L Na-pyruvate, 4 mM L-glutamine, 3.7 g/L NaHCO_3_, 10,000 U/mL penicillin G, 10 mg/mL streptomycin sulfate, and 25 µg/mL amphotericin B supplemented with 0.25% trypsin (Invitrogen). After centrifugation at 1000 *g* and room temperature (RT) for 10 min, the pellet was resuspended, washed in 10 mL of DMEM containing 10% heat-inactivated fetal bovine serum (FBS; Invitrogen), and again centrifuged for 10 min at 1000 *g* and RT. The final pellet was filtered through a sterile filter (100 µm pore size; Greiner Bio-One Hungary Kft., Mosonmagyaróvár, Hungary) to eliminate tissue fragments that had resisted dissociation. The cells were resuspended in 2 mL of the same solution and then seeded on poly-L-lysine-coated culture flasks (75 cm^2^; 10^7^ cells/flask) and cultured at 37 °C in a humidified air atmosphere supplemented with 5% CO_2_. The medium was changed the next day and then every 3 days. After 7 days of culture, microglial cells in the primary cultures were shaken off using a platform shaker (120 rpm for 20 min) at 37 °C as we described earlier [73]. Microglia were collected from the supernatant by centrifugation (3000 *g* for 8 min at RT), resuspended in 4 mL of DMEM/10% FBS, and seeded in the same medium either on poly-L-lysine-coated coverslips (15 × 15 mm; 2 × 10^5^ cells/coverslip) for immunocytochemistry or in poly-L-lysine-coated Petri dishes (10^6^ cells/Petri dish) for western blot analysis. The number of cells collected was determined in a Bürker chamber after trypan blue staining. DMEM/10% FBS was replaced the next day and then on the third and sixth days of subcloning (subDIV6). These cultures were used in previous studies [5,73], and 73.3% ± 17.8% purity was routinely achieved for microglia in secondary cultures [73]. In this latter study, we demonstrated that the main contaminating cell types were glial fibrillary acidic protein-immunoreactive astrocytes (19.0% ± 2.7%), β-tubulin III-positive neurons (3.1% ± 0.4%), and 2′,3′-cyclic nucleotide 3′-phosphodiesterase-positive oligodendrocytes (0.2% ± 0.1%). In the present study, only samples of harvested cultures from different breeding runs with the highest microglia purity (typically around 98%) were selected through immunocytochemical validation as reported earlier [73].

On subDIV6, the expanded microglia-enriched cultures were treated for 24 h with LPS alone (20 ng/mL final conc., dissolved in DMEM; Sigma-Aldrich), KYNA alone (1 µM final conc., dissolved in DMEM), SZR104 alone (1 µM final conc., dissolved in DMEM), or with a combination LPS + KYNA or LPS + SZR104. LPS treatment served as an immunochallenge. The following six culture types were used: (a) control (unchallenged and untreated) cultures, (b) 20 ng/mL LPS-stimulated cultures, (c) 1 µM KYNA-treated cultures, (d) 1 µM SZR104-treated cultures, (e) LPS-challenged + KYNA-treated cultures (at indicated doses), and (f) LPS-challenged + SZR104-treated cultures (at indicated doses).

### 4.4. Immunocytochemistry

The microglia-enriched secondary cultures were assessed using antibodies against a microglia-specific antigen, two inflammation markers, an unmodified core histone H3, and two antigens that recognize specific histone H3 lys modifications (Table S2). Immunocytochemistry was performed according to our previously used protocols [5,73]. Briefly, the cells were fixed in 4% formaldehyde in 0.05 M phosphate-buffered saline (pH 7.4) at RT for 5 min and then rinsed in 0.05 M phosphate-buffered saline for 3 × 5 min. After permeabilization and blocking of the nonspecific sites for 30 min at 37 °C in 0.05 M phosphate-buffered saline containing 5% normal goat serum and 0.3% Triton X-100, the cells on the coverslips were incubated overnight at 4 °C with the appropriate primary antibody diluted in 0.05% phosphate-buffered saline containing 1% bovine serum albumin and 0.3% Triton X-100 solution. The cells were then washed in 0.05 M phosphate-buffered saline for 3 × 5 min at RT before being incubated without Triton X-100 but with the appropriate Alexa Fluor fluorochrome-conjugated secondary antibody in the dark for 2 h at RT. Afterwards, the cells were washed in 0.05 M phosphate-buffered saline for 3 × 5 min and then in distilled water once for 5 min at RT. Lastly, the prepared coverslips were mounted on microscope slides in Prolong Diamond Antifade with 4′,6-diamidino-2-phenylindole dye (DAPI; Thermo Fisher, Waltham, MA, USA). To confirm the specificity of the secondary antibodies, omission control experiments (i.e., staining without the primary antibody) were also carried out. In these experiments, immunocytochemical signals were not observed.

### 4.5. Western Blot Analysis

Cultured cells were collected with a rubber policeman; homogenized in 50 mM Tris-HCl (pH 7.5) containing 150 mM NaCl, 0.1% Nonidet P40, 0.1% cholic acid, 2 µg/mL leupeptin, 1 µg/mL pepstatin, 2 mM phenylmethylsulfonyl fluoride, and 2 mM ethylenediaminetetraacetic acid; and then centrifuged at 10,000 *g* for 10 min. The pellet was discarded, and the protein concentration of the supernatant was determined [74]. Due to the high purity and low yield of the cultures, the protein concentration was typically low (about 0.5 µg/µL). Western blot analysis was performed as previously described [72] with the exception of the occasional use of large, five-well combs to make wells with 50 µL capacity (Mini-Protean Tetra Cell module; Bio-Rad Hungary Ltd., Budapest, Hungary) to accomodate larger volumes in order to compensate for lower protein content; consequently, such sample sets (control and 5 treatments) were often run in two gels. Briefly, equal amounts of proteins in the linear range of detection were loaded onto a polyacrylamide gel. For the quantitative assessment of protein expression on western blots, 10 µg of protein was denatured at 100 °C for 5 min, loaded into wells, and separated on 12% sodium dodecyl sulfate–polyacrylamide gel before being transferred onto a Hybond-ECL nitrocellulose membrane (Amersham Biosciences, Little Chalfont, Buckinghamshire, England), blocked for 1 h in 5% nonfat dry milk in 0.1 M Tris-buffered saline containing 0.1% Tween 20, and finally incubated overnight with the appropriate antibody (Table S2). Nonspecifically bound or excess antibody was removed with 5 × 5 min rinses in 0.1 M Tris-buffered saline containing 0.1% Tween 20. The membranes were then incubated for 1 h with the appropriate peroxidase-conjugated secondary antibody. The enhanced chemiluminescence method (Amersham Biosciences) was used according to the manufacturer’s instructions to reveal immunoreactive bands. Proper dilutions and exposure times for each antibody were tested before performing the actual experiments. GAPDH detection was used as a control for equal protein load.

Grayscale digital images of the blots were acquired by scanning the autoradiographic films with a desktop scanner (Epson Perfection V750 Pro; Seiko Epson Corp., Nagano, Japan). Images were scanned and processed at identical settings to allow comparisons to be made of the blot results obtained from different samples. The densities of immunoreactive lanes equally loaded with protein aliquots were quantified, and data values were presented as a percentage of the control. For statistical comparisons, a one-way ANOVA or Mann–Whitney rank sum test was used and a *p* value of < 0.05 was considered significant. Values are presented as the mean ± standard error of the mean (SEM) from at least five immunoblots, one from each independent experiment.

### 4.6. Image Analysis and Statistics

Digital images were captured using a Leica DMLB epifluorescence microscope equipped with a Leica DFC7000 T CCD camera (Leica Microsystems CMS GmbH, Wetzlar, Germany) and via the LAS X Application Suite X (Leica). For the intracellular (nuclear and cytoplasmic) localization and quantitative analyses of the levels of unmodified core histone H3 and the methylated histone H3 proteins H3K9me3 and H3K36me2, the DAPI-labeled cell nuclei of anti-CD11b/c-labeled cells were identified on coverslip-cultured samples.

For the quantitation of immunofluorescent images, 65–180 randomly selected CD11b/c-positive microglia were analyzed from three separate experiments. A quantitative microscopic analysis of histone immunofluorescence was conducted in ImageJ (version 1.47; originally developed by W. Rasband at the U.S. National Institutes of Health, Bethesda, MD, USA) [75], available at https://imagej.net/Downloads, accessed on 10 July 2013). Briefly, the densities of the whole cell and nuclei (mean gray values), the areas, and their integrated optical densities (fluorescence per area) were calculated. The corrected total cell fluorescence (CTCF) values (CTCF_whole cell_ and CTCF_nucleus_) were then computed as described in the method developed by L. Hammond (Queensland Brain Institute, The University of Queensland, Australia), available at https://theolb.readthedocs.io/en/latest/imaging/measuring-cell-fluorescence-using-imagej.html, accessed on 30 October 2020, as follows: CTCF = integrated density − (area of selected cell × mean fluorescence of background readings). Lastly, cytoplasmic CTCF values were calculated using the following formula: CTCF_cytoplasm_ = CTCF_whole cell_ − CTCF_nucleus_. Identical microscopic and software parameter settings were then applied for each color channel. The color correction of images was occasionally performed when photomicrographs were prepared for publication. Statistical comparisons were made using SigmaPlot, and the data values were analyzed using Kruskal–Wallis one-way ANOVA on ranks followed by Dunn’s method for pairwise multiple comparisons of differences between groups; the significance was set at *p* < 0.05. Data values are presented as the mean ± SEM.

## Data Availability

Data is contained within the article.

## Supplementary Materials

The following are available online at https://www.mdpi.com/article/10.3390/ijms23031079/s1.

## Abbreviations

ANOVA	one-way analysis of variance
CCR1	C-C motif chemokine receptor 1 (also known as chemokine receptor 1)
CD11b/c	cluster of differentiation 11b/c, the rat CR3 complement receptor
CTCF	corrected total cell fluorescence, an area-dependent value
CXCL10	C-X-C motif chemokine ligand 10 (also known as interferon-inducible cytokine IP-10)
DAPI	2-[4-(aminoiminomethyl)phenyl]-1H-indole-6-carboximidamide hydrochloride
DIV	day(s) in vitro
DMEM	Dulbecco’s Modified Eagle’s Medium
FBS	fetal bovine serum
GAPDH	glyceraldehyde 3-phosphate dehydrogenase (EC 1.2.1.12)
H	histone
H3K	histone H3 lys modification
Iba1	ionized calcium-binding adaptor molecule 1
IgG	immunoglobulin G
ICC	immunocytochemistry
KYNA	kynurenic acid
PBS	phosphate-buffered saline
rpm	revolutions per minute
RT	room temperature
SEM	standard error of the mean
subDIV	subcloned day(s) in vitro
SZR104	N-(2-(dimethylamino)ethyl)-3-(morpholinomethyl)-4-hydroxyquinoline-2-carboxamide
TBS	Tris-buffered saline
WB	western blot
