# Supplementary material for: Kynurenic Acid and Its Analog SZR104 Exhibit Strong Antiinflammatory Effects and Alter the Intracellular Distribution and Methylation Patterns of H3 Histones in Immunochallenged Microglia-Enriched Cultures of Newborn Rat Brains
Source: Int J Mol Sci. 2022 Jan 19;23(3):1079. doi: 10.3390/ijms23031079 (PMC8835130; doi:10.3390/ijms23031079)
Supplement: Supplementary file 1 [file ijms-23-01079-s001.zip › Table S1.pdf]

**Table S1.** Molecular structure, chemical name, empirical formula and molecular weight of KYNA and its analog SZR104.

| Abbreviation | Molecular structure | Chemical name | Empirical formula and molecular weight |
|---|---|---|---|
| KYNA | 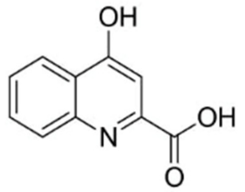 | 4-hydroxyquinolin-2-carboxylic acid | $C_{10}H_7NO_3$<br><br>189.17 |
| SZR104 | 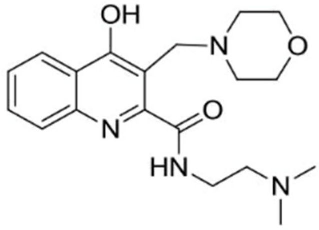 | *N*-(2-(dimethylamino)ethyl)-3-(morpholinomethyl)-4-hydroxyquinoline-2-carboxamide | $C_{19}H_{26}N_4O_3$<br><br>358.43 |
