# Supplementary material for: Kynurenic Acid and Its Analog SZR104 Exhibit Strong Antiinflammatory Effects and Alter the Intracellular Distribution and Methylation Patterns of H3 Histones in Immunochallenged Microglia-Enriched Cultures of Newborn Rat Brains
Source: Int J Mol Sci. 2022 Jan 19;23(3):1079. doi: 10.3390/ijms23031079 (PMC8835130; doi:10.3390/ijms23031079)
Supplement: Supplementary file 1 [file ijms-23-01079-s001.zip › Table S2.pdf]

Table S2. Primary and secondary antibodies used in western blots and immunocytochemistry.

Primary antibody, abbreviated name	Primary antibody, full name	Final dilution (for ICC or WB)	Company	Secondary antibody with fluorochrome, full name	Final dilution (for ICC or WB)	Company
CD11b/c	Mouse anti-CD11b+CD11c ab.	1/500 (ICC)	Abcam, Cambridge, England	Alexa Fluor 568 goat anti-mouse (ICC)	1/1000 (ICC)	Invitrogen, Thermo Fisher Scientific Carlsbad, CA, USA
CXCL10	Rabbit anti- C–X–C motif chemokine ligand 10 polycl. ab.	1/200 (ICC)	Biorbyt, Cambridge, UK	Alexa Fluor 488 goat anti-rabbit (ICC)	1/1000 (ICC)	Invitrogen, Thermo Fisher Scientific Carlsbad, CA, USA
CCR1	Rabbit anti-CCR1 polycl. ab.	1/200 (ICC); 1/250 (WB)	Novus, Centennial, CO, USA	Alexa Fluor 488 goat anti-rabbit (ICC); horseradish peroxidase goat anti-rabbit ab. (WB)	1/1000 (ICC); 1/2000 (WB)	Invitrogen, Thermo Fisher Scientific Carlsbad, CA, USA (ICC); Sigma-Aldrich, Budapest, Hungary (WB)
H3	Rabbit anti-Histone H3 polycl. ab.	1/500 (ICC); 1/1500 (WB)	Abcam, Cambridge, England	Alexa Fluor 488 goat anti-rabbit (ICC); horseradish peroxidase goat anti-rabbit ab. (WB)	1/1500 (ICC); 1/2000 (WB)	Invitrogen, Thermo Fisher Scientific Carlsbad, CA, USA (ICC); Sigma-Aldrich, Budapest, Hungary (WB)
H3K9me3	Rabbit anti-Histone H3 (trimethyl K9) polycl. ab.	1/500 (ICC)	Abcam, Cambridge, England	Alexa Fluor 488 goat anti-rabbit (ICC)	1/1000 (ICC)	Invitrogen, Thermo Fisher Scientific Carlsbad, CA, USA
H3K36me2	Rabbit anti-Histone H3 (dimethyl K36) monocl. ab.	1/500 (ICC)	Abcam, Cambridge, England	Alexa Fluor 488 goat anti-rabbit (ICC)	1/1000 (ICC)	Invitrogen, Thermo Fisher Scientific Carlsbad, CA, USA
GAPDH	Mouse anti-glyceraldehyde 3-phosphate dehydrogenase antibody	1/20000 (WB)	Sigma Aldrich, Budapest, Hungary	Horseradish peroxidase rabbit anti-mouse (WB)	1/2000 (WB)	Sigma-Aldrich, Budapest, Hungary

ICC: immunocytochemistry, WB: Western blot.
